# Parallel Three-Branch Correlation Filters for Complex Marine Environmental Object Tracking Based on a Confidence Mechanism

**DOI:** 10.3390/s20185210

**Published:** 2020-09-12

**Authors:** Yihong Zhang, Shuai Li, Demin Li, Wuneng Zhou, Yijin Yang, Xiaodong Lin, Shigao Jiang

**Affiliations:** College of Information Science and Technology, Engineering Research Center of Digitized Textile & Fashion Technology, Ministry of Education, DongHua University, Shanghai 201620, China; 2191514@mail.dhu.edu.cn (S.L.); deminli@dhu.edu.cn (D.L.); wnzhou@dhu.edu.cn (W.Z.); 2171318@mail.dhu.edu.cn (Y.Y.); 2191511@mail.dhu.edu.cn (X.L.); 2191460@mail.dhu.edu.cn (S.J.)

**Keywords:** parallel three-branch correlation filters, confidence mechanism, complex marine environment, object tracking

## Abstract

Marine object tracking is critical for search and rescue activities in the complex marine environment. However, the complex marine environment poses a huge challenge to the effect of tracking, such as the variability of light, the impact of sea waves, the occlusion of other ships, etc. Under these complex marine environmental factors, how to design an efficient dynamic visual tracker to make the results accurate, real time and robust is particularly important. The parallel three-branch correlation filters for complex marine environmental object tracking based on a confidence mechanism is proposed by us. The proposed tracker first detects the appearance change and position change of the object by constructing parallel three-branch correlation filters, which enhances the robustness of the correlation filter model. Through the weighted fusion of response maps, the center position of the object is accurately located. Secondly, the Gaussian-triangle joint distribution is used to replace the original Gaussian distribution in the training phase. Finally, a verification mechanism of confidence metric is embedded in the filter update section to analyze the tracking effect of the current frame, and to update the filter sample from verification result. Thus, a more accurate correlation filter is trained to prevent model drift and achieve a good tracking effect. We found that the effect of various interferences on the filter is effectively reduced by comparing with other trackers. The experiments prove that the proposed tracker can play an outstanding role in the complex marine environment.

## 1. Introduction

Due to the complexity of the marine environment, it is meaningful to study the tracking of complex marine environmental objects [1]. Owing to changes in appearance, changes in lighting, fast motion, blur and partial occlusion, it is difficult for traditional visual object trackers to achieve accurate and robust tracking of complex marine environmental objects [2]. Therefore, based on the in-depth analysis of the visual object tracking technology, this passage proposes a series of strategies to improve the tracking performance for complex marine environmental objects.

Correlation filtering is a key point of object tracking research, which originated from signal processing [3]. Given the object in the first frame, perform feature extraction on the object and analyze the region of interest. Then, find similar features and regions of interest in subsequent images and make predictions for the position of the next frame. We take the coordinates corresponding to maximum peak as the location of the object. Following this, the earliest tracker, MOSSE [4], was proposed, which exploited the most simple tracking method of related filtering ideas. Subsequently, there were many related improvements based on MOSSE, such as CSK [5], KCF [6], etc. According to introducing nuclear method, they have achieved good results, especially the KCF using the cyclic matrix calculation makes the tracking speed amazing. Subsequently, the DSST [7] tracker innovatively handles scale changes. Two independent correlation filters, the translation filter and scale filter, are used for object positioning and scale evaluation. Due to the problem that all the above-mentioned methods are influenced by the boundary effect. The SRDCF [8] tracker came into being. The tracker takes the image signal as large as possible, retains more real information of the object, and then punishes samples that are farther from the center of the object through a spatial weight coefficient. 

Then the ECO_HC [9] tracker was proposed at the top computer vision conference CVPR2017. This method uses efficient convolution operations to extract the feature matrix and performs multi-feature fusion to obtain the feature map. Correlate the response spectrum and analyze it to get the position of the object. The ECO_HC tracker effectively improves the problems of low filter efficiency and the overfitting of related filters.

However, the ECO_HC tracker also has its shortcomings. When the object occurring deformation, light changes, or self-rotation, it is no reason to renew the relevant model with the same learning rate. In addition, the use of a smooth Gaussian distribution as the expected output will make the positioning inaccurate [10]. Subsequently, the PCF [11] tracker came into being. Compared with the ECO_HC tracker on the video sequences, precision accuracy and overlap accuracy of the PCF tracker are better than the former. When the object rotates significantly, the light changes and deforms and the PCF tracker can successfully track the object. However, the above trackers do not effectively track the object in the complex marine environment specifically. So as to promote the precision of tracking performance in the complex marine environment, we put forward parallel three-branch correlation filters for tracking the object, which can efficiently improve the robustness of object tracking [12]. Aiming at the problem that parallel three-branch correlation filters lack effective supervision mechanism, this paper proposes a confidence mechanism to analyze the distribution of related responses to verify whether it is reliable [13], and supervise the update of the sample model with inspection results.

The contributions of this paper are as follows:

First, target samples with three different weights are respectively trained with three parallel correlation filters and, where the values of the weights are determined by the learning rate. Three different learning rates are used to renew the model and perform weighted fusion to effectively improve the robustness of the tracker to overcome bad conditions in the marine environment.

Second, the confidence metric proposed in this paper judges whether to update the filter, that is, to supervise the update of the sample model. Confidence Response (CR) aims to evaluate the numerical difference of the largest n responses on the relevant response graph. When the CR value is above the threshold, the result is valid and the sample model can be updated, otherwise it will be not updated.

Third, we have done a lot of experiments in the ocean environment, which fully proves that the proposed tracker can still show excellent results when the background is blurred and the waves interfere. In addition, we also compared nine other representative trackers on the OTB-2015 dataset, showing that the proposed tracker can deal with object tracking efficiently in complex and changing scenarios.

## 2. Related Work

The PCF tracker is the baseline tracker of this article, and it has a good effect on object tracking in complex scenes. In the initial frame, the PCF tracker uses the shared generated samples and improved expected output to train two parallel correlation filters. Then, in the present frame, according to balancing the response maps of two filters, the PCF tracker is able to detect the location of the object with the Newton method [14]. Subsequently, the PCF tracker uses Gaussian Mixture Model to add a new sample or merge the two most similar samples to generate a new sample. Then, we use the new sample to renew the PCF1 and PCF2 at different learning rates.

PCF tracker uses object samples $f_{1k}^{l},f_{2k}^{l},\ldots f_{mk}^{l}$ with different weights $w_{kp}^{t}$ to train two parallel correlation filters, $h1_{t}^{l}$ and $h2_{t}^{l}$, respectively. The value of the weights $w1_{kp}^{t}$ and $w2_{kp}^{t}$ are changed according to learning rate. The objective functions of PCF1 and PCF2 are shown in Equations (1) and (2), respectively:

(1) $$\varepsilon (h1_{t})=\sum_{k=1}^{n}{\left\|\sum_{l=1}^{d}h1^{l}*\sum_{p=1}^{m}\left(w1_{kp}^{t}f_{kp}^{l}\right)-gs_{k}\right\|}^{2}+\lambda \sum_{l=1}^{d}{\left\|\omega h1^{l}\right\|}^{2}$$

(2) $$\varepsilon (h2_{t})=\sum_{k=1}^{n}{\left\|\sum_{l=1}^{d}h2^{l}*\sum_{p=1}^{m}\left(w2_{kp}^{t}f_{kp}^{l}\right)-gs_{k}\right\|}^{2}+\lambda \sum_{l=1}^{d}{\left\|\omega h2^{l}\right\|}^{2}$$

where p,k represents the amount of samples generated by training data and cyclic shift method, respectively, and $gs_{k}$ represents the improved joint-expected output distribution, which is a linear weighted fusion of Gaussian distribution and triangular distribution. The second term of the two equations are regularization terms, and ω represents spatial regularization parameters, which are used to prevent the model from overfitting and used to solve boundary effects. The λ is a regularization parameter used to prevent the overfitting of the correlation filtering model. The greater the λ, the greater the punishment. The common methods such as the L-curve and the GCV for solving ill-conditioned equations are compared with the analysis of examples. The calculation example shows that the Tikhonov regularization parameter optimization method is a feasible and effective method to get the optimal regularization parameter λ.

The fast Fourier transform can effectively transform Equations (1) and (2) into the frequency domain for efficient calculation. Since the regularization parameters ω break the closed solution of the function and the solution cannot be directly obtained for the objective equation, the conjugate gradient method can obtain the solution sum of the above two objective functions. Then, the output response score of the relevant filter is calculated by Equation (3), and finally the location of the object is obtained by the Newton iteration method.

(3) $$y_{t,pos}=F^{-1}\left\{\sum_{l=1}^{d}\left(\alpha H1_{t-1}^{l}Z_{t,pos}^{l}+(1-\alpha )H2_{t-1}^{l}Z_{t,pos}^{l}\right)\right\}$$

where α is the fusion factor, and $H1_{t-1}^{l}$ and $H2_{t-1}^{l}$ are two filters in the frequency domain.

After object positioning is completed, the image features of the object are collected from the positioning area as new samples, and then the similarity between the new samples and the existing training set samples is calculated using the Gaussian mixture model. If new samples and the training samples have a particularly big feature difference, the new sample is added and the old sample added previous is removed to make sure that the number of samples remains unchanged. If the similarity between the new sample and the training sample is high, the two most similar samples are merged. The weight of each training sample is updated by Equation (4):

(4) $$w_{kp}^{t}=(1-\eta )w_{kp}^{t-1}$$

where η is the learning rate and t represents the t-th frame. It can be drawn from the above formula that different learning rates determine different sample weights.

To judge the scale of the object, the DCF tracker is used to extract the scale features under different scale factors [15], and then the relevant output response score of each scale factor is calculated using Equation (5). Finally, the scale corresponding of the max output response score is the scale of the object.

(5) $$y_{t,scale}=F^{-1}\left\{\frac{\sum_{l=1}^{d}{\bar{A}}_{t-1,scale}^{l}Z_{t,scale}^{l}}{B_{t-1,scale}+\lambda }\right\}$$

where ${\bar{A}}_{t-1,scale}^{l}$ and $B_{t-1,scale}$ represent the scale correlation filter of the previous frame, and λ(λ≥0) is the regularization parameter.

The expected output of the traditional DCF method generally follows a two-dimensional Gaussian distribution [16], and the peak value is relatively smooth. However, because the DCF model uses a cyclic shift window to generate lots of synthetic training samples, the only real sample is in the center position, so the sharp expected output peak is more reasonable and the drift of the relevant filtering model can be avoided. In the expected output response graph, the areas farther away from the center represent negative sample labels, so the values of these locations should be set to tend to zero, while the triangular distribution graph has a smaller decline rate at the valley. Therefore, Gaussian distribution (as shown in Equation (6)) and triangular distribution (as shown in Equation (7)) are not suitable for DCF tracker. The joint expected output response distribution is used to replace the original Gaussian distribution graph. As shown in Equation (8), the Gaussian distribution and the triangular distribution are organically fused together by a multiplication operator [17], which can promote the robustness of the tracker.

(6) $$g(x,y)=\frac{1}{2\pi \sigma^{2}}e^{-\frac{\left(x^{2}+y^{2}\right)}{2\sigma^{2}}},(-\frac{a}{2}\leq x\leq \frac{a}{2},-\frac{b}{2}\leq y\leq \frac{b}{2})$$

(7) $$s(x,y)=(1-\frac{2}{a}|x|)(1-\frac{2}{b}|y|),(-\frac{a}{2}\leq x\leq \frac{a}{2},-\frac{b}{2}\leq y\leq \frac{b}{2})$$

(8) $$m(x,y)=\frac{1}{2\pi \sigma^{2}}(1-\frac{2}{a}|x|)(1-\frac{2}{b}|y|)e^{-\frac{\left(x^{2}+y^{2}\right)}{2\sigma^{2}}},(-\frac{a}{2}\leq x\leq \frac{a}{2},-\frac{b}{2}\leq y\leq \frac{b}{2})$$

where σ represents the standard covariance of the distribution map, x,y represent the axis of the distribution map coordinates, a,b, respectively, the width and height of the distribution map, and the origin of the coordinates of the distribution map is $\left(\frac{a}{2},\frac{b}{2}\right)$.

## 3. Proposed Approach

### 3.1. Parallel Three-Branch Correlation Filters

The DCF tracking algorithm can effectively train the discriminant related filter model in the frequency domain. The difference between the object and the background is effectively used. When the object is in a more complex scene, the algorithm has better tracking and discrimination capabilities. After the object is located, the target image feature is extracted, and the related filter model is updated online. The formula is as follows:

(9) $$\min_{H^{*}}={\left\|\sum_{l=1}^{d}h^{l}f_{1}^{l}-f_{2}\right\|}^{2}+\lambda \sum_{l=1}^{d}{\left\|h^{l}\right\|}^{2}$$

where l is the dimension, and h is an improved correlation filter. λ is the adjustment parameter to control the influence of regularization [18]. Calculate the partial derivative after FFT and simplify to get:

(10) $$H^{l}=\frac{\bar{G}F_{t}^{l}}{\sum_{k=1}^{d}{\bar{F}}_{t}^{k}F_{t}^{k}+\lambda }$$

where *G*, *F* are after Fourier transform. Calculate the numerator and denominator, respectively, in the process of resolving:

(11) $$y=F^{-1}\left\{\frac{\sum_{l=1}^{d}{\bar{A}}^{l}Z^{l}}{B\;+\;\lambda }\right\}$$

where $F^{-1}$ is the inverse Fourier transform, *A*, *B* represent the numerator and denominator, respectively, and z is the calculated feature map. The value of y is changed by changing the value of z. When y is the largest, it is the time when the correlation filter is most suitable.

(12) $$A_{t}^{l}=(1-\eta )A_{t-1}^{l}+\;\eta F_{t+1}F_{t}^{l}$$

(13) $$B_{t}=(1-\eta )B_{t-1}+\;\eta \sum_{k=1}^{d}{\bar{F}}_{t}^{k}F_{t}^{k}$$

where *η* is the learning rate, and *t* represents the number of frames. From the first frame, neither the numerator nor the denominator has the previous item, and the initial numerator and denominator are well obtained.

Then, the tracker with parallel three-branch correlation filters is proposed by us [19]. The tracker uses object samples $f_{1k}^{l},f_{2k}^{l},\ldots f_{mk}^{l}$ with different weights $w_{kp}^{t}$ to train three parallel correlation filters, $h1_{t}^{l}$, $h2_{t}^{l}$ and $h3_{t}^{l}$, respectively. The value of the weights $w1_{kp}^{t}$, $w2_{kp}^{t}$ and $w3_{kp}^{t}$ are decided by the learning rate. The functions of PCF1, PCF2 and PCF3 can be shown as Equations (14), (15) and (16), respectively:

(14) $$\varepsilon (h1_{t})=\sum_{k=1}^{n}{\left\|\sum_{l=1}^{d}h1^{l}*\sum_{p=1}^{m}\left(w1_{kp}^{t}f_{kp}^{l}\right)-gs_{k}\right\|}^{2}+\lambda \sum_{l=1}^{d}{\left\|\omega h1^{l}\right\|}^{2}$$

(15) $$\varepsilon (h2_{t})=\sum_{k=1}^{n}{\left\|\sum_{l=1}^{d}h2^{l}*\sum_{p=1}^{m}\left(w2_{kp}^{t}f_{kp}^{l}\right)-gs_{k}\right\|}^{2}+\lambda \sum_{l=1}^{d}{\left\|\omega h2^{l}\right\|}^{2}$$

(16) $$\varepsilon (h3_{t})=\sum_{k=1}^{n}{\left\|\sum_{l=1}^{d}h3^{l}*\sum_{p=1}^{m}\left(w3_{kp}^{t}f_{kp}^{l}\right)-gs_{k}\right\|}^{2}+\lambda \sum_{l=1}^{d}{\left\|\omega h3^{l}\right\|}^{2}$$

The conjugate gradient method is used to obtain the sum of the solutions of the above three objective functions [20]. The output response score of the relevant filter is calculated by Equation (17), and finally the position of the object in the present frame is obtained by the Newton iteration method. Three correlation filters use three different learning rates to determine the location of the object.

(17) $$y_{t,pos}=F^{-1}\left\{\sum_{l=1}^{d}\left(\alpha H1_{t-1}^{l}Z_{t,pos}^{l}+\beta H2_{t-1}^{l}Z_{t,pos}^{l}+(1-\alpha -\beta )H3_{t-1}^{l}Z_{t,pos}^{l}\right)\right\}$$

where α, β are fusion factors, and $H1_{t-1}^{l}$, $H2_{t-1}^{l}$ and $H3_{t-1}^{l}$ represent three parallel correlation filters. Three different learning rates are used to renew the model and perform weighted fusion [21] to effectively improve the performance of the tracker for complex marine environmental objects.

The weight of each training sample is updated by Equation (18):

(18) $$w_{kp}^{t}=(1-\eta )w_{kp}^{t-1}$$

Then, the relevant output response score of each scale factor is calculated using Equation (19).

(19) $$y_{t,scale}=F^{-1}\left\{\frac{\sum_{l=1}^{d}{\bar{A}}_{t-1,scale}^{l}Z_{t,scale}^{l}}{B_{t-1,scale}+\lambda }\right\}$$

### 3.2. Confidence Machanism

**Figure 1:**
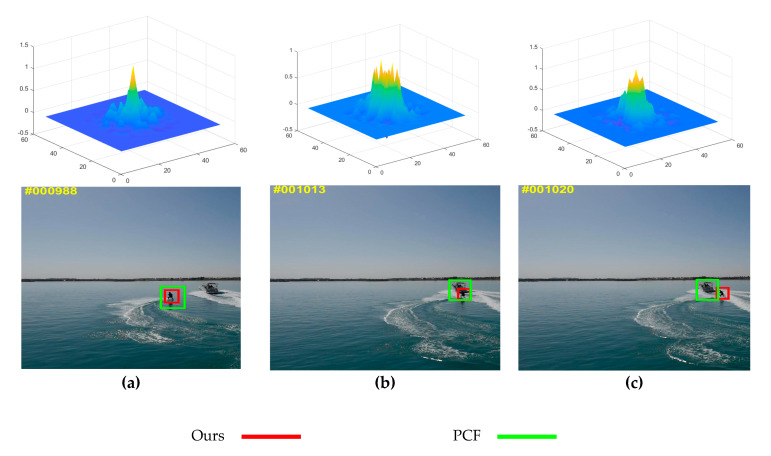
Comparison of the peak distribution of the PCF tracker and the proposed tracker when occurring occlusion. The red one is the proposed tracker and the green one is the baseline tracker PCF. There are three status: (**a**) The object has not affected by interence; (**b**) The object has affected by interence; (**c**) The object gradually breaks away from the interence.

Without an effective supervision mechanism during the tracking of the parallel three-branch correlation filters method, errors are easily generated when the object is interfered with by external environmental factors such as occlusion and illumination changes, and then the errors are brought into the update of the sample model. The accumulated error will cause the tracking frame to drift, which will eventually cause the object to be lost. Aiming at the problem that the method of parallel three-branch correlation filters lacks effective supervision mechanism, this paper proposes a verification mechanism [22] to analyze the reliability of current results by evaluating the distribution of related responses, and supervise the update of the sample model based on the inspection results. Before inputting the tracking object area into the relevant filter, a verification mechanism of confidence measurement is added to analyze and verify the distribution of the relevant response and tracking results, and judge whether to update the sample model according to the verification mechanism [23]. When the tracking result meets the set conditions, it is used to update the sample model, otherwise it is not adopted. The confidence metric in the update stage of the filter acts a pivotal part in tracking the quality of the tracking effect. The traditional tracking method compares the value of the related responses and takes the object area with the highest related response as the correct tracking result. In practical applications, this evaluation method has a large error. When there is an interference area with a high similarity, the object may be lost, but the value of its related response is still high, as shown in Figure 1. When the form of object changes, the relevant response value may be low, which will affect the judgment of the tracking effect.

In most cases, we usually take the largest point of the peak as the object location, which is not reliable [24]. This paper introduces a new confidence metric CR (Confidence Response) and embeds it in the filter update module to analyze the peak distribution of the relevant response and evaluate the tracking results. The solution process is as follows: select n related responses from the current frame image and arrange them in order from largest to smallest. Use R(i) to indicate the response value in the i-th position, p(R(i)) is the probability of the response value appearing. Find the expected μ of n response values:

(20) $$\mu =\sum_{i=1}^{n}R(i)\times p(R(i))$$

Then, introduce the concept of standard deviation to measure the response value R(i). The available CR formula is as follows:

(21) $$CR=\sqrt{{\sum_{i=1}^{n}\left(R(i)-\mu \right)}^{2}}$$

CR aims to evaluate the numerical difference of the largest n responses on the relevant response graph [25]. The larger the CR value, the more dispersed the distribution between the interference area and the true area in the frame, and the more reliable the tracking effect; otherwise, the more concentrated the distribution between the interference area and the true area in the frame, the more unreliable the tracking effect.

As shown in Figure 1, the peak response is generally regarded as the location of the object [26]. However, in reality, when occurring occlusion, deformation, etc., the peak response does not represent the location of the actual object. As shown in Figure 1a, when there is no interference, the response peak is single, which can track the object well. As shown in Figure 1b, when there is interference, the response peak gradually increases. At this time, the highest peak is not the actual object position. As shown in Figure 1c, taking the highest peak position as the object position will cause tracking failure such as object drift [27]. Therefore, the confidence metric proposed in this paper judges whether to update the filter, that is, to supervise the update of the sample model. When the CR value is above the set threshold, the result is valid and the sample model can be updated, otherwise it is not updated. As shown in Figure 1c, when the CR value is lower than the threshold we set, the model update is stopped [28].

When the object is occluded, the baseline method PCF continuously updates the sample model, resulting in the accumulation of errors gradually and the occurrence of tracking drift [29]. The proposed method judges whether to update the sample model with the comparison result of the confidence metric CR value and the threshold value, and the update is no longer allowed when the object is occluded. Therefore, the improved sample update strategy can effectively solve the problem that the baseline PCF method easily causes tracking frame drift and object loss. According to the confidence mechanism, we can effectively improve the performance of the tracker.

### 3.3. Algorithm of the Proposed Tracker

As shown in Algorithm 1, we use three parallel correlation filters with different learning rates to make the tracking more robust. Firstly, if we use more branches, the speed will drop drastically, which makes tracking not time sensitive. Secondly, too many branches will cause overfitting. We combine the confidence mechanism and three-branch correlation filtering to be almost the same as the baseline tracker in terms of rate, but greatly improve the accuracy. The specific algorithm is shown below, which specifically explains the working principle step by step. In the initial video frame, the algorithm uses the prior information of the object to initialize three parallel correlation filters. Firstly, update the position by fusing the values of the three-branch parallel correlation filter, then use the scale filter to calculate the scale of the object, and finally extract the features of the object based on the new position and scale, and use the GMM to generate a new sample set to train the relevant filter model online. Finally, use the confidence mechanism to determine whether the model is updated. In subsequent frames, repeat the above steps.
**Algorithm 1**: Parallel Three-Branch Correlation Filters Tracking Algorithm Based on a Confidence Mechanism**Input:**   **1:** Image $I_{t}$.  **2:** Detected target position $P_{t-1}$ and scale $S_{t-1}$.**Output:**  **1:** Detected target position $P_{t}$ and scale $S_{t}$.**Loop:**  **1:**
*Initialize*
$H1_{1}^{l}$, $H2_{1}^{l}$, $H3_{1}^{l}$
*and*
$A_{1,scale}$, $B_{1,scale}$
*in the first frame by Equations (14),(15),(16) and (12),(13).*  **2: for**
$t\in \left[2,t_{f}\right]$
**do**.  **3:  Position detection:** **  4:**    Extract position features $Z_{t,pos}$
*from*
$I_{t}$
*at*
$P_{t-1}$ and $S_{t-1}$ by a search region.  **5:   ** Compute three parallel correlation scores $y1_{t,pos}$, $y2_{t,pos}$, $y3_{t,pos}$.  **6:   ** Merge the three correlation scores to $y_{t,pos}$
*by Equation (17)*.  **7:   ** Set $P_{t}$ to the target position by Newton iterative method.  **8:  Scale detection:**  **9:**    Extract scale feature $Z_{t,scale}$
*from*
$I_{t}$
*at*
$P_{t-1}$ and $S_{t-1}$
*by a search region*.  **10:**   Compute correlation scores $y_{t,scale}$
*by Equation (19)*.  **11:**   Set $S_{t}$ to the target scale that maximizes $y_{t,scale}$.  **12: Model update:**  **13:  ** Extract new sample features $F_{t,pos}$
*and*
$F_{t,scale}$
*from*
$I_{t}$ and $S_{t}$.  **14:**   Generate new training set by the GMM method.  **15:**   Compute response scores CR
*by Equation (21)*.  **16:**   If CR > threshold, return step 2; else continue.  **17:**   Update the model $H1_{t}^{l}$, $H2_{t}^{l}$, $H3_{t}^{l}$
*by the learning rate*
$\eta_{t1}$, $\eta_{t2}$, $\eta_{t3}$.  **18:**   Update the scale model $A_{t,scale}$, $B_{t,scale}$
*by the learning rate*
$\eta_{0}$.  **19:  Return**
$P_{t}$, $$S_{t}$$.  **20: end for**.

## 4. Results

### 4.1. Experimental Results and Analysis in the Complex Marine Environment

**Figure 2:**
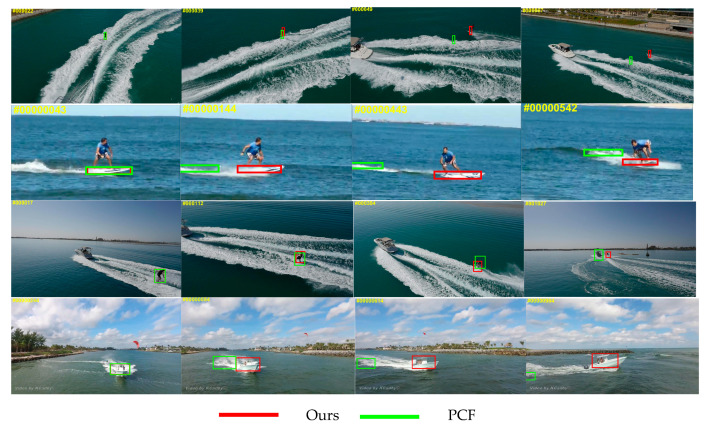
Comparison results of the proposed tracker and the baseline tracker PCF in the complex marine environment.

To prove that our tracker is superior to the baseline tracker, Figure 2 shows the comparison between the baseline tracker PCF and the tracker proposed in this paper in different complex marine environments. The experiments clearly show that the proposed tracker can effectively suppress the drift caused by tracking failure, which makes the tracking effect more robust.

For the purpose of verifying that the proposed tracker can track the complex marine environmental objects with better performance, we conduct a comparative experiment on the complex marine environmental objects to compare the proposed tracker with the current representative trackers, including the PCF tracker [11], ECO-HC tracker [9], Staple tracker [30] and CSK tracker [5]. 

**Figure 3:**
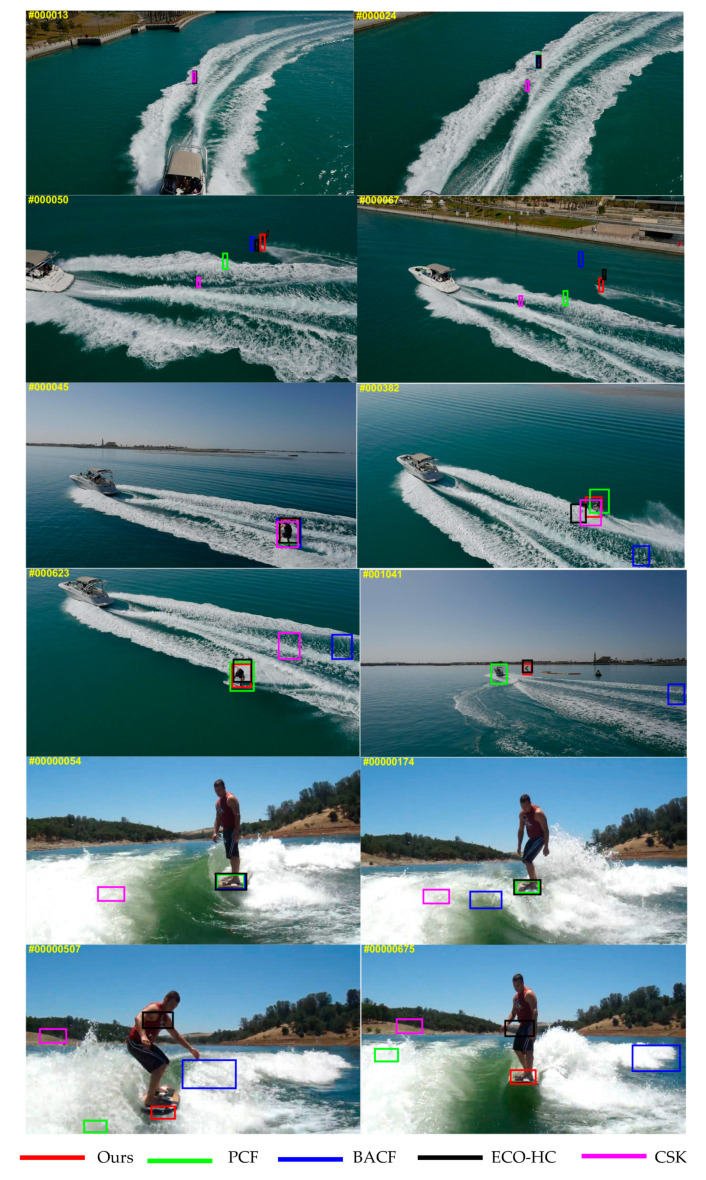
Qualitative experimental results from marine environmental videos. Five different colors represent representative five trackers, respectively.

Figure 3 shows that the proposed tracker behaves significantly better than that of several other trackers when tracking complex marine environmental objects with motion blur and rotation deformation; the proposed tracker can effectively learn changes in appearance and accurately locate the object. The following three video paragraphs are representative, highlighting the target tracking in the three most common marine environments, especially the tracking of small targets in the ocean, the interference of ocean waves, and the interference of other ships. We select a few representative frames of each data, so the selection is meaningful. We can see from several sets of pictures that when other trackers have already drifted (once they drift, they will continue to drift), our trackers can still perform well and robustly, and this is an intuitive experience. We have a lot of data later to verify the excellent performance of our tracker.

### 4.2. Comparison of the Proposed Tracker and the Baseline Tracker on OTB-2013

**Figure 4:**
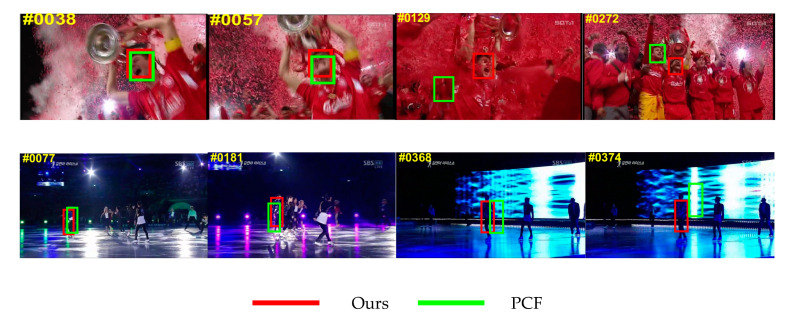
Comparison experimental results of the proposed tracker and the baseline tracker PCF.

We have done experiments on the OTB-2013 dataset [31] to compare the benchmark tracker with the proposed tracker. Table 1 and Table 2 show the center position accuracy and overlap accuracy of the tracker under 11 different attributes, the attributes include scale variation of the object(SV), illumination variation of the environment (IV), rotation of the object out of plane (OPR), the object is occlusion (OCC), the background of the object appears cluttered (BC), the object deforms (DEF), the object produces motion blur (MB), the fast motion of the object (FM), the rotation of the object in the plane (IPR), the movement of the object out of view (OV), and the low resolution of the camera (LR). Compared with the baseline tracker, the tracking accuracy of the proposed tracker has been improved in nine attributes. The results are shown in Figure 4, which further verified that the tracker in this paper can performs better than baseline tracker in complex environment, especially for the following two attributes: the illumination variation of the environment and the background of the object appears cluttered. These attributes determine whether the proposed tracker can perform excellently in the complex marine environment.

### 4.3. Results and Analysis on the Dataset OTB-2015

The OTB-2015 dataset [32] is a benchmark dataset commonly used for object tracking experiments. Compared with the OTB-2013 dataset, the OTB-2015 dataset is a more challenging dataset that includes 100 videos, and also has eleven various attributes. On this very challenging dataset, we do the experiments with other nine trackers: ECO-HC [9], BACF [33], LMCF [34], DSST [7], LCT [35], SAMF [36], Staple [30], SRDCF [8] and KCF [6].

**Figure 5:**
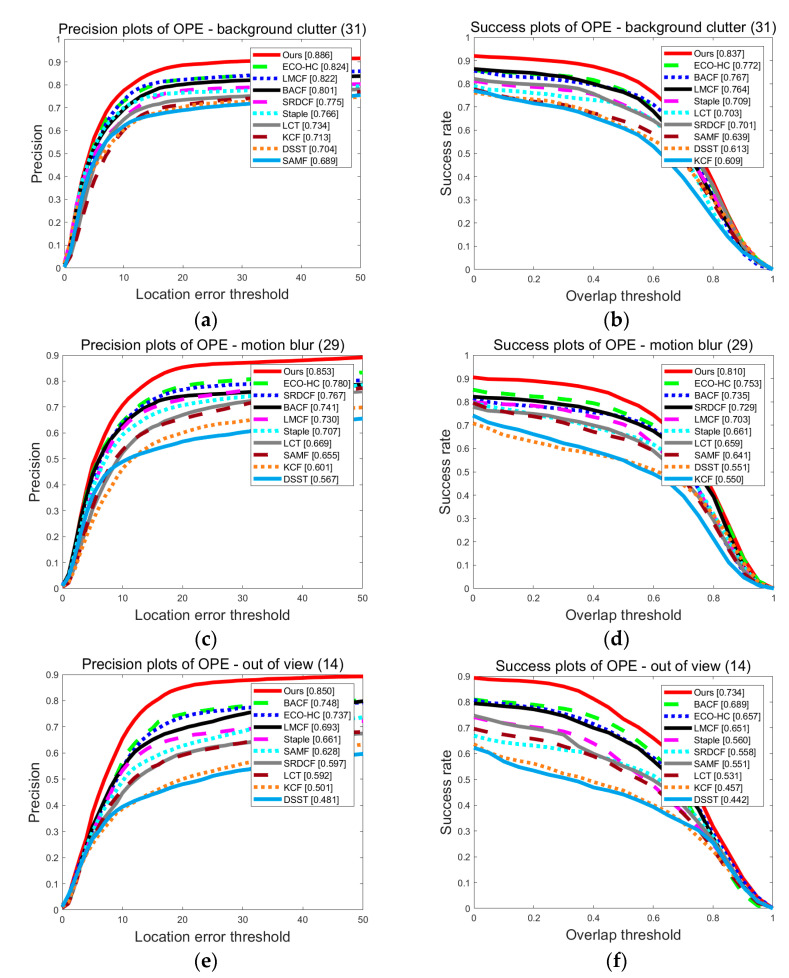
Experimental results show that the proposed tracker performs excellently, particularly in three attributes: (**a**) The PP of the OPE on attribute BC; (**b**) The SP of the OPE on attribute BC; (**c**) The PP of the OPE on attribute MB; (**d**) The SP of the OPE on attribute MB; (**e**) The PP of the OPE on attribute OV; (**f**) The SP of the OPE on attribute OV.

Figure 5 shows the tracking accuracy of each tracker under the three attributes of BC (contains 31 video sequences), MB (contains 29 video sequences) and OV (contains 14 videos). According to the figure, the proposed tracker achieved the highest tracking accuracy among the three attributes, the AP and the AUC under these three attributes were 88.6% and 83.7%, 85.3% and 81.0%, 85.0% and 73.4%. The experiments further verify that the proposed tracker can solve the problem of blurred background and rapid change.

**Figure 6:**
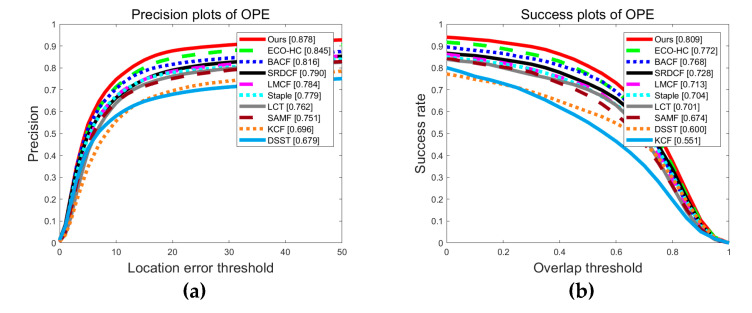
Experimental results for the representative ten trackers represented by ten different color curves: (**a**) The PP of the OPE on all sequences; (**b**) The SP of the OPE on all sequence.

**Figure 7:**
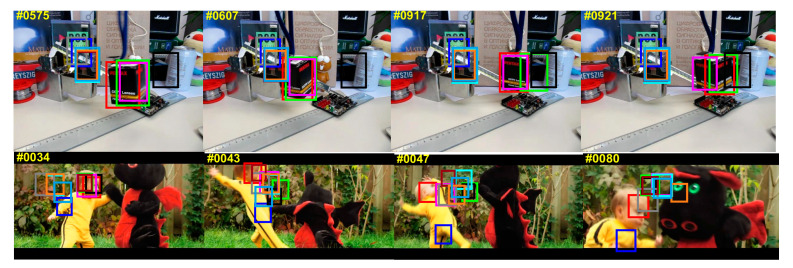

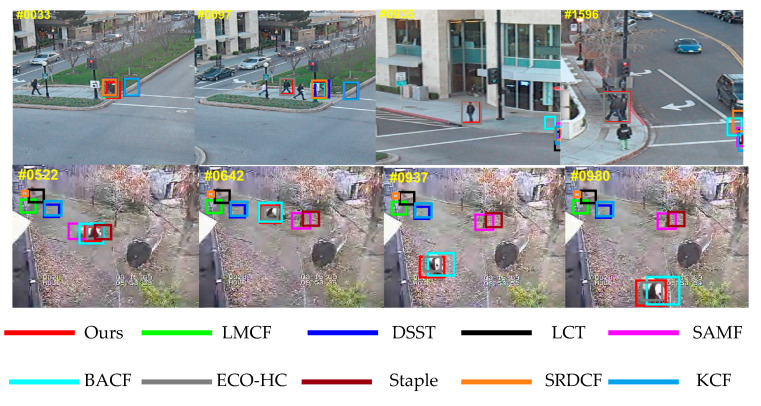
Qualitative experimental results from four representative videos. Ten different colors represent representative ten trackers, respectively.

Figure 6 shows the average accuracy of ten trackers on the OTB-2015 dataset. From the figure, we can see that, among these 10 trackers, the proposed tracker performs best with the AP of 87.8% and the AUC of 80.9% respectively. Besides, this section also evaluates the 10 trackers in 11 attributes. From the Table 3, the proposed tracker has the highest AP among 10 attributes. From Table 4, the proposed tracker achieved the highest AUC among all 10 attributes. In summary, the proposed tracker has significantly improved tracking accuracy, robustness and real-time performance. Besides, we show a qualitative experiment results on the OTB-2015 dataset with four representative video selected sequences. The results are shown in Figure 7. We select a few representative frames of each data so the selection is meaningful. The following video paragraphs are representative, highlighting the object tracking for changes in form, interference from obstructions, changes in light. These factors are particularly important in the marine environment. The proposed tracker has more excellent performance than other nine trackers in the perspective of position estimation and scale estimation, which can deal with changes in the complex marine environment and make the tracking effect outstanding for marine objects.

### 4.4. Failure Case

**Figure 8:**
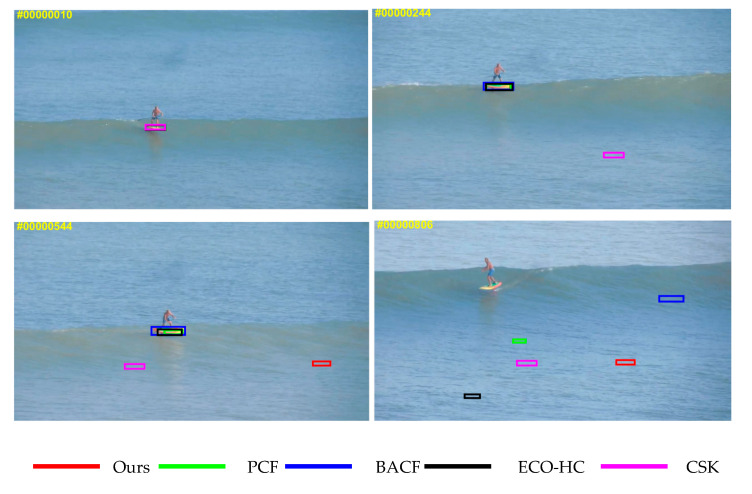
Failure case of proposed tracker on complex marine environmental objects. The results for representative five trackers are expressed in different colors.

Although the proposed tracker can achieve outstanding results in most marine environments, it is easy to lose an object in cases of low resolution and large movements at the same time. We can see from the experimental results in Figure 8 that, in this case, many trackers, including the tracker proposed in this paper, have lost the target, resulting in continuous drift. As part of the next step, we will add a deep-learning neural network, such as the siamese network, to improve the tracker, and effectively improve the effect when adapting to scenarios in various complex environments. The siamese neural network breaks the limitation that the tracker based on the deep neural network cannot be real time. At the same time, the tracker based on the siamese neural network also has high robustness. By calculating the degree of correlation between the candidate domain to be detected and the target area, it is determined that the position with the highest similarity value is the predicted position of the object to be tracked. So, we will add the siamese network to our tracker to make the tracking effect more robust.

## 5. Conclusions 

The baseline tracker PCF is easily interfered with by occlusion, strong light and other factors, which leads to tracking failure. In order to track the complex marine environmental objects efficiently, the parallel three-branch correlation filters based on a confidence mechanism is proposed. Three different parallel correlation filters with three different learning rates are applied to improve the robustness of the tracking effect. In addition, a new tracking performance evaluation index is proposed, and the measurement result is used as a reference index for the filter update. The proposed tracker effectively reduces the interference caused by marine environmental factors, which leads to excellent performance of object tracking. Through the experimental results, we can conclude that the tracker proposed in this paper has outstanding attributes in light changes, interference occlusion, fast motion and scale changes. It can effectively solve the target tracking loss caused by interference from waves and ships, light changes, and fast movement in the complex marine environment. Compared with other trackers, the proposed tracker shows excellent performance with accuracy and success rate. It shows good robustness under the conditions of background changes and its own non-rigid transformation, verifying that the proposed tracker is outstanding in the complex marine environment. However, when the object is blocked and there is a large displacement at the same time, it cannot perform well. Based on the end-to-end feature fusion framework of the siamese network, the framework can effectively fuse CNN features and hand-designed features, solve the problem of parameter learning in feature fusion, and improve the versatility of the target tracker. So, we will try to improve the tracker with the siamese network in the future.

## Figures and Tables

**Table 1:** Central position accuracy and average accuracy of the proposed tracker and baseline tracker in 11 different attributes. Red represent the better performance.

Trackers	SV	IV	OPR	OCC	BC	DEF	MB	FM	IPR	OV	LR	AP
Baseline	83.5	82.3	87.0	90.1	84.5	91.7	76.8	77.7	81.2	81.0	53.3	88.0
Ours	85.9	84.9	88.6	92.2	87.8	91.6	79.6	81.4	83.3	80.1	53.9	89.3

**Table 2:** Overlap accuracy and average accuracy of the proposed tracker and baseline tracker in 11 different attributes. Red represent the better performance.

Trackers	SV	IV	OPR	OCC	BC	DEF	MB	FM	IPR	OV	LR	AUC
Baseline	77.1	78.5	79.6	84.7	80.5	91.0	76.8	75.3	72.8	82.8	53.9	82.5
Ours	77.7	79.4	79.9	85.3	81.6	90.7	76.8	75.6	73.2	81.9	55.3	82.9

**Table 3:** Central position accuracy and average accuracy of representative ten trackers in 11 different attributes. Red, green, and blue, respectively, represent the top three trackers.

Trackers	SV	IV	OPR	OCC	BC	DEF	MB	FM	IPR	OV	LR	AP
**Ours**	84.3	84.0	86.5	84.8	88.6	84.3	85.3	82.5	82.3	85.0	79.0	87.8
ECO-HC	80.5	79.2	81.1	80.6	82.4	81.8	78.0	79.2	78.3	73.7	79.8	84.5
BACF	76.7	80.3	77.9	73.0	80.1	76.4	74.1	78.7	79.2	74.8	74.1	81.6
SRDCF	74.1	78.6	74.2	73.0	77.5	72.8	76.7	76.9	74.5	59.7	65.5	79.0
LMCF	72.3	79.5	76.0	73.6	82.2	72.9	73.0	73.0	75.5	69.3	67.9	78.4
Staple	71.5	78.7	73.0	72.1	76.6	74.3	70.7	69.7	77.0	66.1	63.1	77.9
LCT	67.8	74.3	74.6	67.9	73.4	68.5	66.9	68.1	78.2	59.2	53.7	76.2
SAMF	70.1	70.8	73.9	72.2	68.9	68.0	65.5	65.4	72.1	62.8	68.5	75.1
KCF	63.5	72.4	67.7	63.2	71.3	61.9	60.1	62.1	70.1	50.1	56.0	69.6
DSST	63.3	71.5	64.4	58.9	70.4	53.3	56.7	55.2	69.1	48.1	56.7	67.9

**Table 4:** Overlap accuracy and average accuracy of representative ten trackers in 11 different attributes. Red, green, and blue, respectively, represent the top three trackers.

Trackers	SV	IV	OPR	OCC	BC	DEF	MB	FM	IPR	OV	LR	AUC
**Ours**	75.4	81.0	77.8	78.3	83.7	77.6	81.0	76.0	72.2	73.4	65.4	80.9
EOC-HC	71.2	75.4	72.3	74.4	77.2	73.4	75.3	74.0	67.7	65.7	69.4	77.2
BACF	69.8	78.0	70.9	69.2	76.7	69.1	73.5	75.6	71.1	68.9	66.3	76.8
SRDCF	66.2	74.0	66.4	67.8	70.1	65.9	72.9	71.7	66.2	55.8	62.6	72.8
LMCF	62.2	74.5	67.7	68.7	76.4	65.9	70.3	67.4	65.6	65.1	54.6	71.3
Staple	61.0	72.1	64.6	67.2	70.9	67.2	66.1	63.8	67.3	56.0	49.1	70.4
LCT	58.3	71.5	67.6	63.1	70.3	61.6	65.9	65.5	69.4	53.1	43.6	70.1
SAMF	58.4	64.0	66.0	66.4	63.9	60.6	64.1	59.5	64.1	55.1	51.5	67.4
DSST	52.5	64.9	55.1	53.1	61.3	47.9	55.1	51.7	58.9	45.7	44.2	60.0
KCF	41.5	55.0	52.7	51.2	60.9	50.3	55.0	52.6	55.3	44.2	29.5	55.1

## References

[1] Chen X., Xu X., Yang Y., Wu H., Tang J., Zhao J. (2020). Augmented Ship Tracking Under Occlusion Conditions From Maritime Surveillance Videos. IEEE Access.

[2] Singh M., Khare S., Kaushik B.K. (2020). Performance Improvement of Electro-Optic Search and Track System for Maritime Surveillance. Def. Sci. J..

[3] Kalal Z., Mikolajczyk K., Matas J. (2012). Tracking-learning-detection. IEEE Trans. Pattern Anal. Mach. Intell..

[4] Bolme D.S., Beveridge J.R., Draper B.A., Lui Y.M. Visual Object Tracking Using Adaptive Correlation Filters. Proceedings of the 2010 IEEE Computer Society Conference on Computer Vision and Pattern Recognition (CVPR).

[5] Henriques J.F., Caseiro R., Martins P., Batista J. (2012). Exploiting the Circulant Structure of Tracking-by-Detection with Kernels. Lect Notes Comput Sc.

[6] Henriques J.F., Caseiro R., Martins P., Batista J. (2014). High-Speed Tracking with Kernelized Correlation Filters. IEEE Trans. Pattern Anal. Mach. Intell..

[7] Danelljan M., Hger G., Khan F.S., Felsberg M. (2017). Discriminative Scale Space Tracking. IEEE Trans. Pattern Anal. Mach. Intell..

[8] Danelljan M., Hager G., Khan F.S., Felsberg M. Learning Spatially Regularized Correlation Filters for Visual Tracking. Proceedings of the 2015 IEEE International Conference on Computer Vision (ICCV).

[9] Danelljan M., Bhat G., Khan F.S., Felsberg M. ECO: Efficient Convolution Operators for Tracking. Proceedings of the IEEE Conference on Computer Vision and Pattern Recognition (CVPR).

[10] Aftab W., Hostettler R., De Freitas A., Arvaneh M., Mihaylova L. (2019). Spatio-Temporal Gaussian Process Models for Extended and Group Object Tracking With Irregular Shapes. IEEE Trans. Veh. Technol..

[11] Yang Y., Zhang Y., Li D., Wang Z. (2019). Parallel Correlation Filters for Real-Time Visual Tracking. Sensors.

[12] Lu X., Li J., He Z., Liu W., You L. (2020). Visual object tracking via collaborative correlation filters. Signal Image Video Process..

[13] Fang Y., Ko S., Jo G.S. (2019). Robust visual tracking based on global-and-local search with confidence reliability estimation. Neurocomputing.

[14] Tjaden H., Schwanecke U., Schömer E., Cremers D. (2018). A Region-Based Gauss-Newton Approach to Real-Time Monocular Multiple Object Tracking. IEEE Trans. Pattern Anal. Mach. Intell..

[15] Yuan Y., Chu J., Leng L., Miao J., Kim B.-G. (2020). A scale-adaptive object-tracker with occlusion detection. Eurasip J. Image & Video Process..

[16] Guo Y., Li Y., Xue A., Tharmarasa R., Kirubarajan T. (2020). Simultaneous tracking of a maneuvering ship and its wake using Gaussian processes. Signal Process..

[17] Zheng H., Tang Y. (2020). A novel failure mode and effects analysis model using triangular distribution-based basic probability assignment in the evidence theory. IEEE Access.

[18] Zhang Q. (1997). Using Wavelet Network in Nonparametric Estimation. IEEE Trans. Neural Netw..

[19] Mercorelli P. (2007). Biorthogonal wavelet trees in the classification of embedded signal classes for intelligent sensors using machine learning applications. J. Frankl. Inst..

[20] Mahdavi-Amiri N., Shaeiri M. (2020). A conjugate gradient sampling method for nonsmooth optimization. A Q. J. Oper. Res..

[21] Gao J., Wang Q., Xing J.L., Ling H.B., Hu W.M., Maybank S. (2020). Tracking-by-Fusion via Gaussian Process Regression Extended to Transfer Learning. IEEE Trans. Pattern Anal. Mach. Intell..

[22] Gao Y., Zhao J., Luo J., Zhou H. (2019). Adaptive feature fusion with the confidence region of a response map as a correlation filter tracker. Opt. Precis. Eng..

[23] Vojir T., Noskova J., Matas J. (2014). Robust Scale-Adaptive Mean-Shift for Tracking. Pattern Recognit. Lett..

[24] Sun H., Chen X., Xiao H. (2019). A deep object tracker with outline response map. CAAI Trans. Intell. Syst..

[25] Li X., Zhou J., Hou J., Zhao L., Tian N. (2020). Research on improved moving object tracking method based on ECO-HC. J. Nanjing Univ. Nat. Sci..

[26] Oron S., Bar-Hillel A., Levi D., Avidan S. (2014). Locally Orderless Tracking. Int. J. Comput. Vis..

[27] Han Y., Deng C., Zhao B., Tao D. (2019). State-Aware Anti-Drift Object Tracking. IEEE Trans Image Process..

[28] Li B., Xie W., Zeng W., Liu W. (2019). Learning to Update for Object Tracking With Recurrent Meta-Learner. IEEE Trans. Image Process..

[29] Lin Y., Chen J., Lin K. Integration of Texture and Depth Information for Robust Object Tracking. Proceedings of the 2014 IEEE International Conference on Granular Computing (GrC).

[30] Bertinetto L., Valmadre J., Golodetz S., Miksik O., Torr P.H. Staple: Complementary Learners for Real-Time Tracking. Proceedings of the 2016 IEEE Conference on Computer Vision and Pattern Recognition (CVPR).

[31] Wu Y., Lim J., Yang M.H. Online Object Tracking: A Benchmark. Proceedings of the 2013 IEEE Conference on Computer Vision and Pattern Recognition.

[32] Wu Y., Lim J., Yang M.-H. (2015). Object Tracking Benchmark. IEEE Trans. Pattern Anal. Mach. Intell..

[33] Galoogahi H., Fagg A., Lucey S. Learning background-aware correlation filters for visual tracking. Proceedings of the 2017 IEEE International Conference on Computer Vision (ICCV).

[34] Wang M., Liu Y., Huang Z. Large Margin Object Tracking with Circulant Feature Maps. Proceedings of the 2017 IEEE Conference on Computer Vision and Pattern Recognition (CVPR).

[35] Ma C., Yang X., Zhang C., Yang M.H. Long-Term Correlation Tracking. Proceedings of the 2015 IEEE Conference on Computer Vision and Pattern Recognition (CVPR).

[36] Li Y., Zhu J. A Scale Adaptive Kernel Correlation Filter Tracker with Feature Integration. Proceedings of the IEEE European Conference on Computer Vision Workshops (ECCVW).

